# Treadmill Exercise Modulates Intestinal Microbes and Suppresses LPS Displacement to Alleviate Neuroinflammation in the Brains of APP/PS1 Mice

**DOI:** 10.3390/nu14194134

**Published:** 2022-10-05

**Authors:** Shunling Yuan, Jialun Yang, Ye Jian, Yong Lei, Sisi Yao, Zelin Hu, Xia Liu, Changfa Tang, Wenfeng Liu

**Affiliations:** 1 Hunan Provincial Key Laboratory of Physical Fitness and Sports Rehabilitation, Hunan Normal University, Changsha 410012, China; 2 Key Laboratory of Protein Chemistry and Developmental Biology of Ministry of Education, Hunan Normal University, Changsha 410081, China

**Keywords:** Alzheimer’s disease, treadmill exercise, neuroinflammation, gut microbiome

## Abstract

Neuroinflammation occurs throughout the pathogenesis of Alzheimer’s disease (AD). Here, we investigated the effects of treadmill exercise on neuroinflammation in APP/PS1 transgenic AD mice and the potential involvement of microbe–gut–brain axis (MGB) mechanisms based on growing evidence that AD’s pathogenesis is correlated with a deterioration in the function of gut microbiota. APP/PS1 transgenic AD mice were subjected to 12 weeks of treadmill exercise, followed by spatial memory tests. After the behavioral study, the amyloid (Aβ) pathology, gut microbes and metabolites, bacterial lipopolysaccharide (LPS) displacement, and degree of neuroinflammation were analyzed. We found that this strategy of exercise enriched gut microbial diversity and alleviated neuroinflammation in the brain. Notably, exercise led to reductions in pathogenic bacteria such as intestinal *Allobaculum*, increases in probiotic bacteria such as *Akkermansia*, increased levels of intestine–brain barrier proteins, and attenuated LPS displacement. These results suggest that prolonged exercise can effectively modulate gut microbes and the intestinal barrier and thereby reduce LPS displacement and ultimately alleviate AD-related neuroinflammation.

## 1. Introduction

Alzheimer’s disease (AD) is the most common neurodegenerative disease in the world [1]. Commonly known as Alzheimer’s disease or cognitive impairment in the elderly, it is characterized by progressive memory impairment, cognitive dysfunction, personality changes, language impairment, and other neuropsychiatric symptoms that affect normal life and eventually lead to disability or even death [2,3,4]. Epidemiological surveys show that the number of existing cases of all forms of dementia worldwide exceeded 57.4 million in 2019 and is expected to reach 152 million by 2050 [5,6]. With the increase in the aging population, AD has become a critical public health issue. The main pathological features of AD are abnormal aggregates of amyloid β-protein (Aβ) plaques and neurogenic fibrillary tangles in addition to microglial hyperactivation, neuronal loss, and the dysfunction of synapses [7,8]. However, the pathogenesis of AD has not been completely elucidated. Currently, treatments centered on its pathological features have failed. Recent studies have revealed that neuroinflammation is the only pathophysiological mechanism present throughout an AD pathogenesis, and it is strongly associated with the peripheral gut microbiota [9,10,11]. Considering the interactions of the various systems throughout the body in AD, reducing neuroinflammation may be a new way to effectively treat AD.

Recently, the brain and gut have been considered as a network called the “microbe–gut–brain axis”, and the gut microbiota has influenced the progress in AD research [12]. There are approximately 100 trillion commensal microorganisms in the human gastrointestinal tract, forming a microecosystem that can influence the brain’s immune homeostasis through the microbe–gut–brain axis and plays a key role in the pathogenesis of neurodegenerative diseases [13]. Considerable evidence shows that with the effects of aging, external stress, etc., there are changes in intestinal and brain barrier permeability, and lipopolysaccharides (LPS) from intestinal microorganisms are more readily displaced into the internal environment, causing the immune activation of the brain through the microbe–gut–brain axis [13,14]. Schoultz et al. [15] demonstrated that AD patients show signs of increased intestinal permeability and dysfunction with elevated levels of LPS in the blood. However, LPS are a specific component of the cell wall of Gram-negative bacteria in the intestine and a strong activator of immune cells, which can cause an inflammatory cascade response [14]. Previously, we performed transcriptome sequencing using an APP/PS1 double transgenic classical AD mouse model and found that the expression levels of genes related to brain tissue barrier function were significantly downregulated in AD mice compared with wildtype mice. Therefore, the function of the intestinal barrier and that of the blood–brain barrier (BBB) may be severely disrupted in AD [8]. Khan et al. [16] showed that LPS activation of Toll-like receptor 4 (TLR4), an immune cell in the intestinal wall, causes intestinal wall inflammation, which leads to intestinal leakage. LPS entering the bloodstream can further disrupt the blood–brain barrier and trigger an inflammatory response by activating TLR4 on the cell membrane while in contact with microglia in the brain [17]. Hence, any intervention aimed at preventing or treating intestinal microecological dysregulation and reducing LPS production may interrupt or slow down the vicious cycle of LPS–neuroinflammation–neurodegeneration.

Numerous studies have shown that exercise has promising preventive and ameliorative effects as an intervention strategy in systemic-onset AD [18,19]. However, it is not clear what role gut microbes play in the process of improving AD through exercise, and most studies have only been conducted on individual tissues or organs; thus, there is little definitive evidence to confirm whether there are interactions between the different systems. From the perspective that exercise has systemic effects, we explored the potential of microbe–gut–brain axis mechanisms to improve the learning and memory abilities in AD subjects through exercise.

## 2. Materials and Methods

### 2.1. Animals

A total of 24 SPF-grade 3-month-old male APP/PS1 double transgenic mice were purchased from Changzhou Cavins Laboratory Animals Co., Ltd. (Changzhou, China; license number: SCXK (Su) 2016-0010) and 24 SPF-grade 3-month-old male C57BL/6J mice were purchased from Changsha Tianqin Biotechnology Co., Ltd. (Changsha, China; license number: SCXK (Xiang) 2019-0014). APP/PS1 mice were randomly divided into an AD calm group (ADC) and AD exercise group (ADE). A total of 24 C57BL/6J mice were randomly divided into a wildtype calm group (WTC) and wild-type exercise group (WTE). The mice had free access to food and water and were housed in an environment with 12 h of light alternating with darkness and a constant temperature of 22–25 °C. The experimental procedures were approved by the professional committee of ethology of Hunan Normal University.

### 2.2. Exercise Intervention Scheme

**Figure 1:**
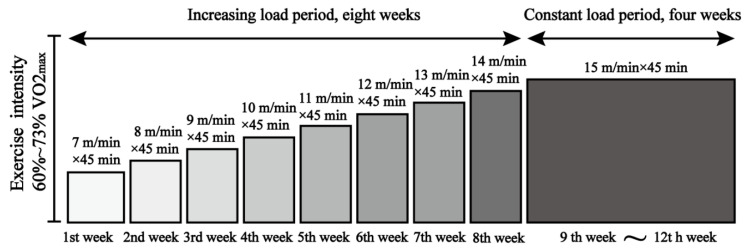
Exercise scheme. The WTE group and ADE group performed treadmill exercise; exercise was performed 5 days a week, with Thursday and Sunday off, within a fixed time window of 5:00–6:00 p.m. for 45 min each time; the WTC and ADC groups were placed on a stationary treadmill during the exercise group training time.

Appropriate levels of exercise are beneficial to the health of an organism, whereas excessive exercise can be harmful [20,21,22]. According to the principle of exercise-related physiological adaptation and overload [23] and referring to the study of Fernando et al. [24] on exercise intensity in mice, we designed a 12-week treadmill exercise scheme with gradual increases in exercise intensity (Figure 1). The WTE group and ADE group performed treadmill exercise, and the speed was incrementally increased from 7 to 14 m/min, with a constant speed of 15 m/min for weeks 9–12 following Schefer et al.’s [25] study, and with a load intensity of 60% to 73% of the maximum oxygen uptake (VO_2_max), representing moderate-intensity aerobic exercise.

### 2.3. Morris Water Maze Experiment

The Morris water maze was used to evaluate the learning and memory abilities of mice. The water maze was 114 cm in diameter and 50 cm in height, with white walls and a water depth of 25 cm. A circular platform of 10 cm in diameter and 24 cm in height was placed in the first quadrant, and the video acquisition system was located directly above the pool. Experiments were conducted daily from 6:00 p.m. to 12:00 a.m., with a constant water temperature of 25 °C. The water was colored white with white nontoxic water-soluble pigments to prevent the mice from seeing the platform during the experiment. The time the mice took to climb the platform (i.e., the latency period) was recorded, and the mice were allowed to stay on the platform for 10 s. If the mice failed to find the platform within 60 s, they were guided to stay on the platform for 10 s. At this point, the latency period was recorded as 60 s. After the positioning navigation experiment, the platform was removed, and the number of times the mice crossed the platform position in the pool, the swimming time and distance within the platform quadrant, and the total swimming distance were observed and recorded within 60 s. Each experiment was completed from the first quadrant to the fourth quadrant in one run.

### 2.4. Material Collection

After the Morris water maze experiment, mice were fasted for 12 h with free access to water. Isoflurane anesthesia was applied the next morning; then, blood was taken from the heart using a syringe containing sodium heparin and centrifuged. The supernatant was aspirated and stored at −80 °C for ELISA. Rectal fecal balls were taken, placed into sterile freezing tubes, immediately immersed into liquid nitrogen, and subsequently stored in a −80 °C freezer until being processed for intestinal microbial and metabolite detection. Brain tissues and intestinal tissues of 1 cm length at 2 cm posterior to the cecum were taken from mice into a freezing tube, immediately immersed in liquid nitrogen, and then stored in a −80 °C freezer until being processed for real-time PCR and Western blotting experiments. Brain and intestinal tissues of four mice in each group were randomly selected and fixed in 4% paraformaldehyde solution for 24–48 h, and sections were then prepared for HE staining as well as immunohistochemistry and immunofluorescence detection.

### 2.5. Nissl Staining

The fixed brain tissue was collected and embedded in 3% agarose, and 40 μm sections were made using a Leica VT1000 S vibrating microtome, placed onto slides, and dried overnight at 37 °C. After rehydration in double-distilled water, Nisin staining solution containing 5% toluidine blue was applied with 1% glacial acetic acid fractionation until Nisin was dark blue with a colorless background. This was followed by alcohol dehydration, and neutral xylene gum was then applied to the sections. Images were acquired using a KEYENCE microscope (Osaka, Japan), and the nisin-staining area was analyzed by ImageJ (NIH, Bethesda, MD, USA).

### 2.6. HE Staining

Routine dehydration, transparency, wax immersion, and embedding were performed to make 5 μm sections, which were dewaxed and hydrated, hematoxylin/eosin-stained, dehydrated, and transparently sealed.

### 2.7. ELISA Assay

IL−22, TNF−α, and LPS ELISA kits (cat.no.203059M, 202412M, 202425M; Ruixin Biotechnology, Quanzhou, China) were used according to instructions. The absorbance (OD) values were measured at 450 nm using an enzyme standard meter, and a standard curve was established to calculate the contents of IL−22, TNF−α, and LPS in each group of mice. The detection method of these kits is a double antibody sandwich enzyme-linked immunosorbent assay. Although LPS from different microorganisms have different biological activities, we expressed the concentration of LPS in the samples as an overall potency.

### 2.8. 16S rDNA Sequencing

Total genomic DNA was extracted from the samples using the CTAB/SDS method. The primers 341F (5′-CCTACGGGRBGCASCAG-3′) and 806R (5′-GGACTACNNGGGTATCTAAT-3′) were used for PCR amplification of the V3–V4 variable region. Sequencing was performed on the NovaSeq 6000 platform from Illumina (Illumina, Tiburon, CA, USA).

### 2.9. Liquid Chromatography–Mass Spectrometry Detection

First, 100 mg of sample was weighed before adding grinding beads and 1 mL of water (containing 0.5% phosphoric acid and 50 μg/mL of internal standard 2-ethylbutyric acid). The sample was ground for 3 min using a freeze grinder (50 Hz), sonicated for 30 min in an ice water bath, left for 30 min at 4 °C, and centrifuged for 15 min at 13,000× *g* at 4 °C. The supernatant was mixed well with 500 μL of ethyl acetate for extraction by vortexing. The sample was ultrasonicated in an ice water bath for 10 min and centrifuged at 4 °C at 13,000× *g* for 10 min before collecting the supernatant for analysis. An Agilent Technologies Inc. (Santa Clara, CA, USA, 8890B-5977B GC/MSD) gas chromatograph was used.

### 2.10. Real-Time PCR Assay

Total RNA was extracted from mouse brain tissue (approximately 30 mg) using TRIzol reagent according to the instructions provided by ThermoFisher Scientific (Thermo Fisher, Waltham, MA, USA). Reverse transcription was performed using miniamp PCR according to the instructions of TransGen Biotech Reverse Transcription Kit (TRAN, Beijing, China); the 20 μL amplification system was configured according to the instructions of the Servicebio qPCR Kit (Servicebio, Wuhan, China), with the BIO-RAD Real-Time PCR system((CFX96; Bio-Rad Laboratories, Hercules, CA, USA) used to complete the amplification. Lastly, according to the detected Ct value, the relative expression of the target gene’s mRNA was calculated using the 2^−∆∆CT^ method with GAPDH as the internal reference gene, and the data were normalized for graphing after statistical analysis. Table 1 shows the primer sequences of target genes.

### 2.11. Immunohistochemical Assays

Two brain vibratome sections were cut at the same location 100 μm apart, and then placed in a 12-well plate. The samples were washed with TBS solution (NaCl 8.8 g + Tris 12.12 g in double-distilled water with concentrated HCl concentration to 1 L; pH = 7.4) for 3 × 5 min, soaked in 3% hydrogen peroxide–methanol solution for 30 min, washed in TBS solution for 5 min, soaked with formic acid with 88% concentration for 7 min, washed with TBS solution for 2 × 5 min, soaked with TBS-A solution (1 L TBS solution + 1 mL Tritonx-100) for 15 min, soaked with TBS-B solution (100 mL TBS-A + 2 g BSA) for 30 min, and incubated with Aβ primary antibody solution (1:1000, 6E10-Mouse IgG, Biolegend, San Diego, CA, USA) overnight at 4 °C.

The samples were rinsed with TBS-A solution for 2 × 5 min, rinsed with TBS-B solution for 15 min, incubated with secondary antibody for 1 h at room temperature, washed with TBS-A solution for 2 × 5 min, washed with TBS-A solution for 15 min, incubated with ABC solution (Vector, Tokyo, Japan, Cat: PK-4002) for 1 h at room temperature, washed with TBS solution 3 × 5 min, and soaked with DAB solution for 2 min 30 s, before the observation of sample staining under a stereomicroscope. The samples were washed with TBS solution for 3 × 5 min, placed on slides with a brush, observed under a stereomicroscope, adjusted to ensure that they were not folded, dried overnight at room temperature, and then processed and sealed using conventional dehydration and transparency methods. Images were acquired using a KEYENCE microscope (Osaka, Japan) and the Aβ-staining area was analyzed by ImageJ (NIH, Bethesda, MD, USA).

### 2.12. Immunofluorescence Assay

Brain vibratome sections were washed in 24-well plates with PBS, and sections were closed with 10 mL of 0.2% TritonX-100 and 150 μL of sheep serum protein for 2 h. Primary antibodies were incubated overnight at 4 °C. Primary antibodies included Iba−1 (1:100, AiFang, Changsha, China), GFAP (1:100, Proteintech, Wuhan, China), TNF−α (1:100, Proteintech, Wuhan, China), and IL−1β (1:100, ABclonal, Wuhan, China).

Sections were rinsed three times with PBS, incubated for 2 h at room temperature, and protected from light following the addition of secondary antibodies conjugated with Alexa Fluor 488 goat anti-rabbit (1:500, Beyotime, Shanghai, China) or goat anti-mouse cy3 (1:500, Beyotime, Shanghai, China). Sections were rinsed three times with PBS, attached to slides, dried in an oven at 37 °C for 5 min, and sealed with a sealer containing DAPI (SouthernBiotech, Birmingham, AL, USA). The images were observed and acquired using a Leica M205 fluorescence microscope. ImageJ (NIH, Bethesda, MD, USA) was used to count the percentage coverage of Iba-1 and GFAP in the area of positive staining in the field of view. The quantitative results concerning IL-1β and TNF-α were expressed as the cumulative fluorescence intensity in the field of view, and to intuitively observe the changes, the WTC group was used as a reference for normalized plotting.

### 2.13. Western Blotting Assay

First, 6 mice were selected from each group, and 30 mg of brain tissue from each of the mice was extracted and added to 300 μL of a mixture of RIPA lysate (Servicebio, Wuhan, China) and 3 μL of cocktail protein inhibitor (CME, Shanghai, China) for homogenization and centrifugation. The supernatant was obtained, followed by the estimation of protein concentration using a BCA protein concentration assay kit (Beyotime, Shanghai, China). The samples were then sequentially sampled, electrophoresed, transferred to membranes, sealed, and incubated with primary antibodies for ZO−1 (1:1000, Proteintech, Wuhan, China), occludin (1:1000, abcam, Cambridge, UK), claudin−5 (1:1000, abcam, Cambridge, UK), and β-actin (1:50,000, abclonal, Wuhan, China) overnight at 4 °C. The next day, the samples were washed with PBST for 3 × 10 min, incubated with secondary antibodies (1:1000, Proteintech, Wuhan, China), and shaken at room temperature for 1 h 20 min. The samples were washed with PBST for 3 × 10 min and imaged using the ECL luminescence kit (Servicebio, Wuhan, China) in a Tanon-5200 gel system to capture pictures. The integrated grayscale values of the samples were analyzed using ImageJ software, and the relative protein expression levels were quantified.

### 2.14. Statistical Analysis

All data are expressed as the mean ± standard deviation (mean ± SD); results were statistically and graphically plotted using GraphPad Prism 8.0 software (GraphPad Software, Inc., San Diego, CA, USA). Statistics were analyzed by two-way ANOVA, followed by multiple comparisons and the least significant difference (LSD) method; *p* < 0.05 indicates a significant difference, and *p* < 0.01 indicates a highly significant difference.

## 3. Results

### 3.1. Exercise Improves the Performance of Learning Memory for Mice

**Figure 2:**
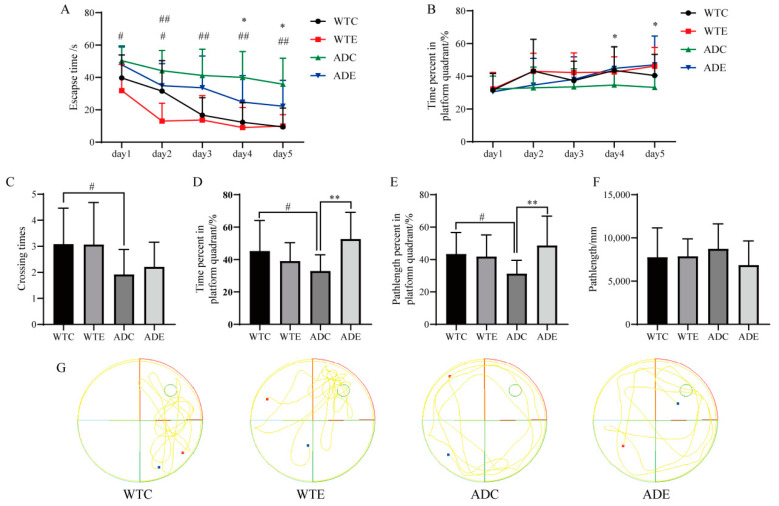
Treadmill exercise improves spatial memory. (**A**) Escape time of the mice in each group during the 5-day learning period. (**B**) Platform quadrant time percentage during the 5-day learning period. (**C**) Number of platform crossings after platform’s removal. (**D**) Platform quadrant percentage time after platform’s removal. (**E**) Platform quadrant percentage distance after platform’s removal. (**F**) Total swimming distance after platform’s removal. (**G**) Swimming path trajectory after platform’s removal. The area enclosed by the red line indicates the platform quadrant, and the small green circle in this quadrant indicates the platform. The red point is the swimming start point, the blue point is the end point, and the yellow line between both points is the swimming path. Data are presented as the mean ± SD (*n* = 12 per group). # *p* < 0.05, ## *p* < 0.01 vs. WTC mice; * *p* < 0.05, ** *p* < 0.01 vs. ADC mice.

In all the groups of mice, there was a decrease in the latency period and an increase in the percentage of time spent in the plateau quadrant as the experiment progressed (Figure 2A,B). During all 5 days, the latency was significantly higher for the ADC group than for the WTC group (*p* < 0.05); when the experiment proceeded to days 4 and 5, the latency significantly decreased (*p* < 0.05) and the platform quadrant time ratio significantly increased (*p* < 0.05) in the ADE group compared with the ADC group. These data suggest that AD mice had an impaired learning ability, and that exercise has a beneficial effect on the learning ability of AD mice, which is consistent with a previous study [26]. Following the platform’s removal, the number of times crossing the platform, the percentage of time in the platform quadrant, and the percentage of distance traveled in the platform quadrant were significantly lower in the ADC group compared with the WTC group (*p* < 0.05); the percentage of time in the platform quadrant and the percentage of distance traveled in the platform quadrant were significantly lower in the ADE group compared with the ADC group (*p* < 0.01), while the number of times crossing the platform increased, but there was no significant difference (*p* > 0.05) (Figure 2C–E), which may be related to the smaller platform area. Notably, there was no significant difference in the total distance of swimming among the four groups of mice, and exercise did not affect the swimming ability of the mice. These data suggest that the spatial memory ability of the AD mice improved after treadmill exercise.

### 3.2. Exercise Reduces Aβ Pathology and Neuronal Loss in Mice

**Figure 3:**
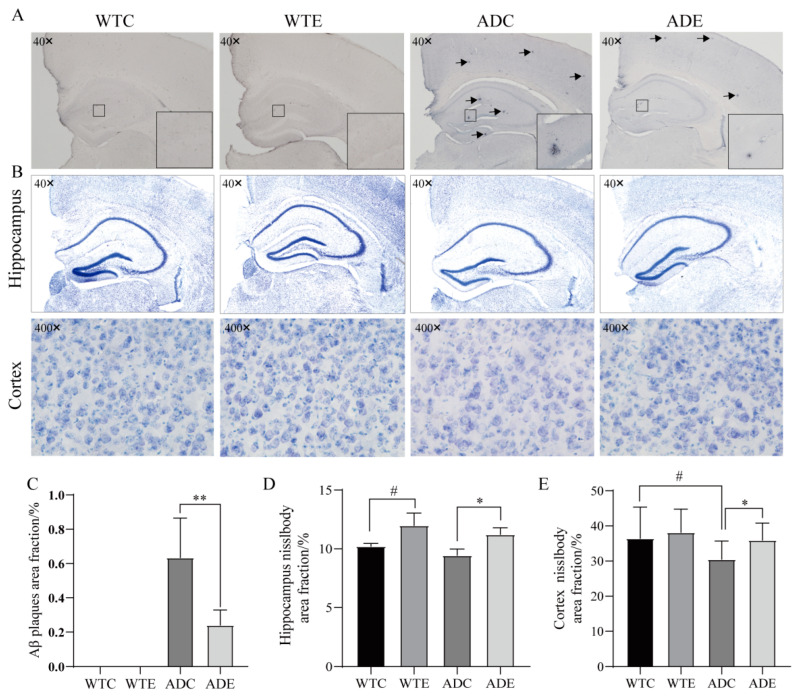
Exercise alleviates Aβ pathology and neuronal loss in mice. (**A**) Representative images of Aβ immunohistochemistry. The black arrows point to Aβ plaques. (**B**) Representative images of Nissl staining. The neuron Nissl body is shown in blue. (**C**) Histogram showing the percentage of Aβ-stained area. (**D**,**E**) Histograms showing the percentage of Nissl-staining area in the (**D**) hippocampus and (**E**) cerebral cortex (*n* = 4 per group). Data are presented as means ± SDs. # *p* < 0.05 vs. WTC mice; * *p* < 0.05, and ** *p* < 0.01 vs. ADC mice.

The understanding of the AD pathogenesis remains incomplete, though the deposition of Aβ plaques in the brain is considered a major pathological hallmark of AD [26,27,28]. The accumulation of Aβ, which leads to neuronal atrophy and death, was once considered a key target for the treatment of AD due to its neurotoxic nature [29]. We analyzed the area of Aβ localization via the area of Nisin staining, and there was no Aβ aggregation in the brains of the WTC and WTE groups, while the ADC and ADE groups showed Aβ aggregation with blue-black spots (Figure 3A). Aβ aggregation was significantly reduced (*p* < 0.01) in the ADE group compared with the ADC group. As can be seen in Figure 3B, the hippocampal niosomes are densely arranged in each group of mice, and the niosomes in the cerebral cortex of the WTC, WTE, and ADE groups are more darkly stained and densely arranged, while the niosomes in the ADC group are more lightly stained, cavernous, and sparsely arranged, which is consistent with recently reported results [30]. Compared with the WTC group, the area of hippocampal niosomes in the WTE group increased significantly (*p* < 0.05), and the area of cortical niosomes in the ADC group decreased significantly (*p* < 0.05); compared with the ADC group, the area of hippocampal and cortical niosomes in the ADE group both increased significantly (*p* < 0.05).

Aβ deposition has been shown to lead to the emergence of many pathologies such as endoplasmic reticulum stress, mitochondrial dysfunction, oxidative stress, and microglial activation, ultimately leading to neuronal loss [31]. Reducing Aβ accumulation was once considered the main goal in the treatment of AD. However, an increasing number of studies have found that neuronal synaptic and neuronal functional impairment in the brains of AD model animals precedes Aβ plaque formation, and pharmacological treatments used clinically for Aβ have repeatedly failed [32]. Therefore, the pathogenesis of AD may be mediated by factors other than Aβ. Therefore, although our experiments show that treadmill exercise significantly reduces neuronal loss in the brains of AD mice, the mechanisms need to be explored more deeply and extensively.

### 3.3. Significant Effect of Treadmill Exercise on Intestinal Microorganisms

Gut microbes play a crucial role in human development, physiology, immunity, and nutrition, which affects not only gastrointestinal health but also brain function and behavior, with the gut even being referred to as the “second brain” [33,34,35]. Numerous studies have found a decrease in gut microbial richness and diversity, an increase in harmful bacteria, and a decrease in beneficial bacteria in AD patients compared with normal individuals of the same age [36]. Therefore, we focused on gut microbes and explored the effect of treadmill exercise on gut microbes.

The α-diversity indices of ACE, Chao, and Shannon in response to species diversity within the sample (Table 2) showed the following order, WTE group > ADE group > WTC group > ADC group, and exercise increased the diversity of intestinal microorganisms in the mice. Compared with the WTC group, the lower α-diversity indices of ACE, Chao, and Shannon in the ADC group indicate that the intestinal microbial diversity is lower in AD model mice than in wildtype mice, consistent with the study of Liu et al. [37]. After exercise, the values of the ACE, Chao, Shannon, and Simpson indices showed greater variation in the AD group than in the WT group, indicating that there was a more significant effect of treadmill exercise on the AD group mice.

**Figure 4:**
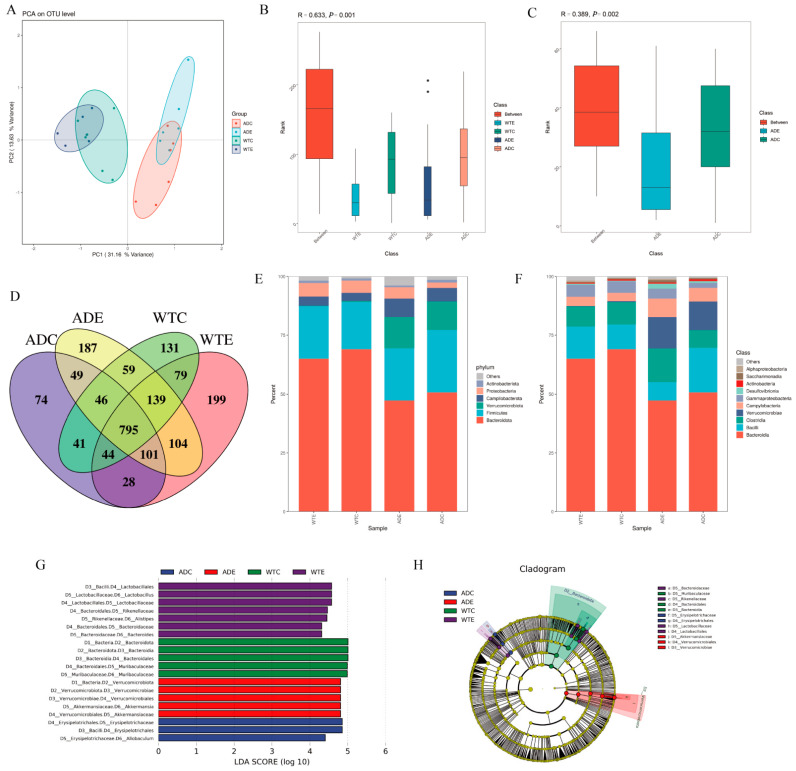
Effects of treadmill exercise on gut microbes. (**A**) PCA cluster analysis indicating overall similarity between mouse gut microbiomes. (**B**,**C**) ANOSIM analysis assessing overall similarity of gut microbes between (**B**) four groups of mice and (**C**) the ADC and ADE groups. *R* > 0 indicates that the difference between groups is greater than the differences within a group, and *p* < 0.01 indicates that there is a significant difference; small black dots indicate possible outliers within the group. (**D**) Venn diagram of OTU at the species level. (**E**) Abundance of species at the phylum level. (**F**) Species abundance at the class level. (**G**) Histogram showing linear discriminant analysis (LDA) scores for significantly altered gut microbes according to LEfSe analysis. Microorganisms with an LDA score ≥4 are shown in the figure. (**H**) Phylogenetic tree cladogram generated by LEfSe analysis showing the phylogenetic distribution of microbiota from phylum to species (*n* = 6 per group).

β diversity was applied to reflect the differences in diversity between samples, and the WTE and ADE groups showed tighter intragroup aggregation, while the WTC and ADC groups had more dispersed intragroup aggregation (Figure 4A). The similarity of the intragroup microbial structure and composition was higher for the exercise group than for the calm group, suggesting that exercise could reduce the differences between individuals within groups to a certain extent. There was a significant difference in the intestinal microbial composition between the four groups of mice (*p* < 0.01) and between the ADC and ADE groups (*p* < 0.02) (Figure 4B,C).

The number of OTUs shared among the four groups was 795, accounting for the majority of the total. The WTE group had the highest number of unique OTUs (199), the ADC group had the lowest (74), and the ADE and WTC groups had 187 and 131 unique OTUs, respectively (Figure 4D). The AD model mice had lower intestinal bacterial flora richness than that of the wildtype mice but were more strongly affected by the treadmill exercise. In addition, the number of OTUs shared between the AD model mice and the WTC group increased after exercise. This indicates that exercise can change the structural composition of the intestinal microbes of mice, increase the number of unique species, and enrich species diversity, prompting the intestinal microbial composition of AD mice to become more similar to that of wildtype mice. At the phylum level (Figure 4E), the dominant phyla in all four groups of mice were *Bacteroidetes* and *Firmicutes*, which accounted for the majority of overall species, which is consistent with the current findings [37]. The abundance of *Bacteroidetes* and *Proteobacteria* was lower in the AD model mice than in the wildtype mice, and exercise downregulated *Bacteroidetes* abundance. At the class level (Figure 4F), the intestinal microbiota of the mice in each group contained mainly *Bacteroidia*, *Clostridia*, *Bacilli*, and *Verrucomicrobiae*. Among them, *Verrucomicrobiae* was the dominant class in the AD mice, and exercise decreased the abundance of *Bacilli* in the AD mice. These data suggest that exercise enriches the intestinal microbial community composition in mice.

The dominant differential bacterial species were screened by an LEfSe analysis with a comparison of the four groups of mice. The dominant species in the WTC group of mice were *Bacteria*, *Bacteroidota*, *Bacteroidia*, *Bacteroidales*, *Muribaculaceae*, and *Muribaculaceae*; the dominant species in the WTE group of mice were *Bacilli*, *Lactobacillales*, *Lactobacillacea*, *Lactobacillus*, *Bacteroidales*, *Rikenellaceae*, *Alistipes*, *Bacteroidales*, *Bacteroidaceae*, and *Bacteroides*; and the dominant species in the ADC group of mice were *Bacilli*, *Erysipelotrichales*, *Erysipelotrichaceae*, and *Allobaculum*. The dominant species in the ADE group of mice were *Bacteria*, *Verrucomicrobiota*, *Verrucomicrobiae*, *Verrucomicrobiales*, *Akkermansiaceae*, and *Akkermansia* (Figure 4G,H). *Akkermansia* is a symbiotic bacterium of the mucus layer that uses mucin as the only source of carbon, nitrogen, and energy, which has metabolic regulatory, immune regulatory, and intestinal health-protective effects [38]. It has been shown that the direct consumption of *Akkermansia* upregulates the intestinal expression of ZO−1 and occludin and effectively thickens the intestinal mucosal barrier [39]. Therefore, treadmill exercise may protect the intestinal barrier and contribute to beneficial effects on the brain by upregulating *Akkermansia* abundance.

### 3.4. Treadmill Exercise Protects the Intestinal Barrier

**Figure 5:**
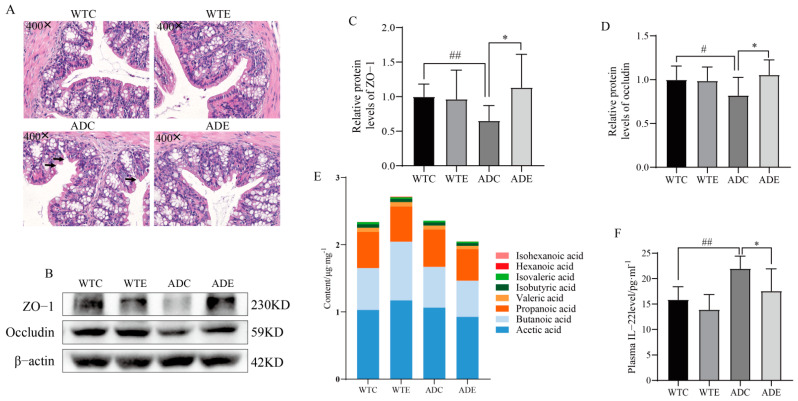
Treadmill exercise protects intestinal barrier. (**A**) Representative images of colonic HE staining. The black arrow indicates where the intestinal barrier is damaged. (**B**–**D**) Relative colon protein contents of (**C**) ZO−1 and (**D**) occludin. (**E**) Species composition and abundance of SCFAs in gut contents. (**F**) The content of serum IL−22 (*n* = 6 per group). Data are presented as means ± SDs. # *p* < 0.05, and ## *p* < 0.01 vs. WTC mice; * *p* < 0.05 vs. ADC mice.

*Akkermansia* can counteract mucosal barrier dysfunction and tight junction protein expression by increasing the level of mucus-producing cup cells, thereby boosting mucus production and maintaining mucosal thickness [40,41], which facilitates the enhancement of intestinal epithelial barrier function. Therefore, we further focused on the changes in intestinal barrier function. A colonic HE staining (Figure 5A) showed that the intestinal mucosa of the ADC group had multiple severe ruptures, and the mucosal layer was thin, sparse, and porous. The cells in the mucosal layer of the ADE group were neatly arranged, dense, smooth, and intact. Further analysis of permeability-related protein expression showed that abundances of colonic occludin and ZO−1 proteins were significantly lower in the ADC group (*p* < 0.05 or *p* < 0.01) compared with the WTC group and were significantly increased by exercise in the AD mice compared with mice of the ADC group (*p* < 0.05) (Figure 5C,D). These data support a protective effect of exercise on the intestinal barrier.

Notably, the SCFAs in the intestine have a nonnegligible role in intestinal permeability. The breakdown and fermentation of undigested dietary carbohydrates in the colon by intestinal microbiota can produce metabolites (SCFAs) that modulate the immune response, reduce systemic inflammation, and maintain the normal function of the intestinal barrier [42]. They can protect the intestine from inflammation by entering CD4^+^ T cells and lymphoid ILCs via G protein receptor-coupled receptor 41 (GPR41) and upregulating IL−22 production [43]. However, we found no significant differences in each group of mice concerning intestinal SCFAs; exercise downregulated the total intestinal SCFAs in AD mice, and the serum IL−22 was significantly elevated in the ADC group, whereas exercise significantly downregulated IL−22 levels (Figure 5E–F). These data suggest that exercise protects the intestinal barrier in mice and may be associated with an increase in intestinal Akkermansia but not SCFAs.

### 3.5. Exercise Attenuates Displacement of LPS to Alleviate Neuroinflammation in the Brains of AD Mice

**Figure 6:**
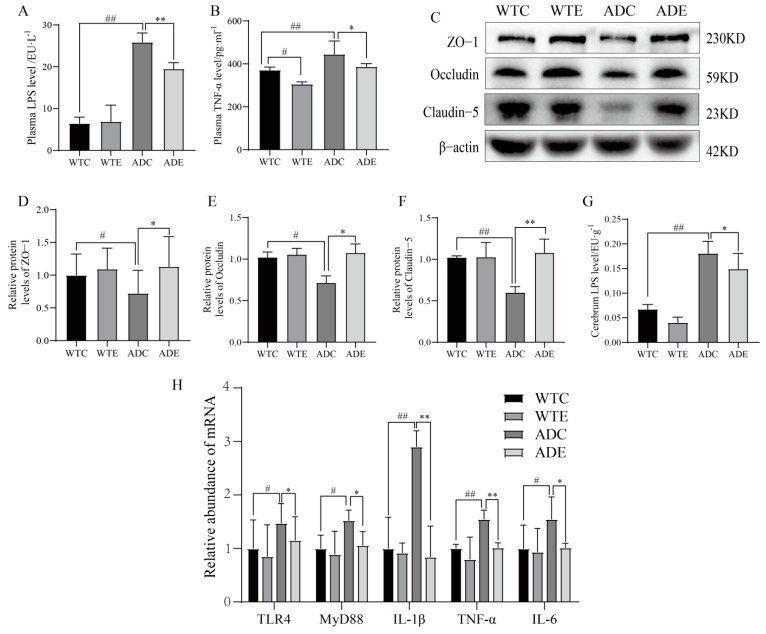
Treadmill exercise inhibits brain leakage and transcription of inflammatory factors. (**A**) Serum LPS level (*n* = 6 per group). (**B**) Serum TNF−α level (*n* = 6 per group). (**C**–**F**) Relative brain protein abundances of (**D**) ZO−1, (**E**) occludin, and (**F**) claudin-5 (*n* = 6 per group). (**G**) Brain tissue LPS levels (*n* = 4 per group). (**H**) Relative mRNA abundance of brain tissue inflammatory pathway factors (*n* = 6 per group). Data are presented as means ± SDs. # *p* < 0.05, and ## *p* < 0.01 vs. WTC mice; * *p* < 0.05, and ** *p* < 0.01 vs. ADC mice.

Since exercise had a dramatic effect on the intestinal barrier in AD mice, we further investigated the displacement of LPS, which are components of the outer wall layer of Gram-negative bacteria in the intestinal microbiota that normally act as a physical barrier providing the bacteria with protection [44]. When the intestinal microbiota is disturbed, large numbers of LPS are dislodged from the cell wall and can enter the internal environment through the intestinal tight junctions [45]. Our results show that serum LPS and TNF−α were significantly elevated (*p* < 0.01), brain LPS were significantly elevated (*p* < 0.01), and the abundances of blood–brain barrier proteins occludin, ZO−1, and claudin−5 were reduced (*p* < 0.05 or *p* < 0.01) in AD mice compared with wildtype mice, trends that were reversed by exercise (Figure 6A–G), which attenuated the occurrence of intestinal and cerebral leaks. When LPS enter the bloodstream from the gut, they activate TLR4 transmembrane recognition receptors on macrophage membranes, leading to the subsequent release of a series of inflammatory factors, which further act in conjunction with LPS, thus altering BBB permeability and exacerbating LPS entry into the brain [14]. These data are consistent with the effect of exercise on the intestinal barrier and support the idea that exercise protects the intestinal barrier, reduces LPS displacement, and further protects the BBB.

**Figure 7:**
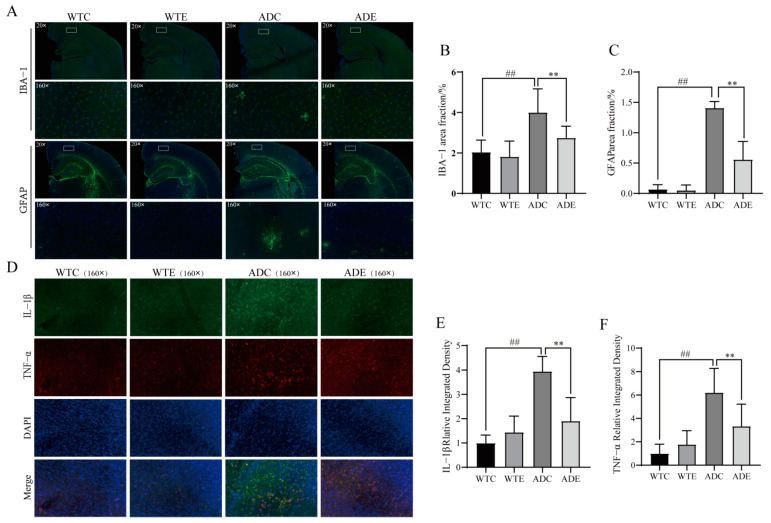
Treadmill exercise reduces glial cell activation and neuroinflammation. (**A**) Representative images of brain Iba−1 and GFAP immunofluorescence. (**B**,**C**) Histograms showing the percentage of (**B**) Iba−1 and (**C**) GFAP areas in brain tissue sections. (**D**) Representative images of brain inflammatory factor immunofluorescence. (**E**,**F**) Histograms showing the percentage of (**E**) IL-1β and (**F**) TNF−α areas in brain tissue sections (*n* = 4 per group). Data are presented as means ± SDs. ## *p* < 0.01 vs. WTC mice; ** *p* < 0.01 vs. ADC mice.

LPS are markers for the invasion of bacterial pathogens that are recognized by the immune system, which initiates the inflammatory response, and they are the most common and important trigger of inflammation [46]. The LPS that reach the brain parenchyma are recognized by TLR4 expressed on microglia [47], which leads to the release of inflammatory factors via the TLR4/MyD88 signaling pathway. We explored this process and found that microglia Iba-1 in the cerebral cortex of AD mice accumulate in a tree-root pattern, while astrocyte GFAP aggregate in a snowflake pattern of abnormal activation (Figure 7A). The inflammatory signaling pathway was thus activated with a significant upregulation of the underlying inflammatory factors (Figure 6G), and exercise reduced neuroglial activation, inhibited the pathway, and downregulated mRNA and the protein expression of inflammatory factors (Figure 6H and Figure 7D). In conclusion, these data further confirm that exercise attenuates the displacement of LPS and ameliorates neuroinflammation in the brains of AD mice via the TLR4/MyD88 signaling pathway.

## 4. Discussion

Physical exercise has a notable effect on the brain and is beneficial for memory health. We observed the effects of treadmill exercise on the spatial learning-memory capacity and brain neuroinflammation in APP/PS1 double transgenic AD mice and investigated the underlying mechanisms of intestinal microbiology. The main findings are as follows: (1) treadmill exercise improves spatial memory capacity in AD mice; (2) exercise reduces Aβ pathology and neuronal loss in AD mice; (3) treadmill exercise has a dramatic effect on intestinal microbes in AD mice, enriching intestinal microbial diversity, reducing pathogenic bacteria such as *Allobaculum*, and increasing probiotic bacteria such as *Akkermansia*; (4) exercise protects the intestinal barrier in AD mice, probably by increasing *Akkermansia* and not SCFAs; and (5) exercise reduces LPS displacement in AD mice, decreases glial cell activation, and alleviates neuroinflammation.

AD neuropathology develops over years to decades, and cognitive dysfunction is a characteristic clinical behavioral feature [48]. Patients with AD who engage in long-term physical exercise demonstrate improved memory capacity [49,50,51]. Our research provides evidence for the beneficial effects of long-term physical exercise on the spatial learning memory capacity and its underlying mechanisms in APP/PS1 mice. In the water maze, the mice in the ADC group showed higher latencies, indicating impaired learning in AD mice; mice in the ADE group showed significantly shorter latencies and a significantly higher percentage of time spent in the platform quadrant on days 4 and 5 compared with mice in the ADC group, indicating that the 12-week exercise intervention significantly improves the learning ability of AD mice. After the removal of the platform, the number of times crossing the platform, the percentage of time in the platform quadrant, and the percentage of distance crossed were significantly lower in the ADC group compared with the WTC group, indicating that the memory ability of AD mice was impaired, while these measurements were higher in the ADE group than in the ADC group, indicating that the 12-week exercise intervention significantly improves the memory ability of AD mice. APP/PS1 mice have learning memory deficits, and we found that exercise significantly inhibits their learning memory decline, which is consistent with the findings of numerous current studies [26,52]. Therefore, long-term exercise can be an effective means of preventing and improving memory loss of AD sufferers.

Clinical studies have shown a significant reduction in the diversity of the intestinal microbiota in AD patients, with an overgrowth of harmful bacteria and a decrease in probiotic bacteria [53]. Ling et al. [54] studied the intestinal microbiota of 100 Chinese AD patients and 71 age- and sex-matched cognitively normal individuals and found significant structural changes in the fecal microbiota of AD patients, with a significant reduction in the alpha and beta diversity indices. Cattaneo et al. [55] found that the abundance of the proinflammatory intestinal microbes (*Escherichia*/*Shigella*) was increased, and the abundance of the anti-inflammatory bacterial flora (*E. rectale*) was reduced in patients with cognitive impairment and cerebral amyloidosis, closely influencing the peripheral inflammatory state. Animal experiments have shown that compared with wildtype mice, APP/PS1 mice begin to show alterations in the intestinal microbiota after 3 months of age, which become more pronounced after 6 months, with a high enrichment of intestinal microbiota associated with inflammation preceding the appearance of key pathological AD features such as brain Aβ deposition and microglial activation [56,57]. Therefore, we used 6-month-old APP/PS1 transgenic mice to assess the relationship between the intestinal microbiota and an AD brain pathology. Numerous studies have shown that intestinal microbial species diversity is one of the main parameters reflecting intestinal microbial stability, homeostasis, and resilience, and that a diverse intestinal microbiota is beneficial for health and is associated with improved learning memory and behavioral flexibility; conversely, low diversity favors ecological dysbiosis, which in turn is strongly associated with disease [58,59,60]. A normal mucosal layer and intestinal barrier can only be maintained in such environments if there is a positive balance between intestinal microbial diversity and between probiotic and pathogenic bacteria [61]. Therefore, the findings in our experiments, wherein treadmill exercise increases intestinal microbial species diversity in mice, support the idea that exercise is beneficial for the homeostasis of intestinal microbial balance, protecting the intestinal barrier and preventing harmful toxins from entering the internal environment. In addition, it is worth exploring the potential mechanisms by which exercise alters the intestinal microbiota. Firstly, moderate exercise may affect the health of the intestinal system through intestinal immune function and intestinal barrier integrity [62]; secondly, exercise affects the enteric nervous system and intestinal motility, as well as the time of intestinal content transport, by stimulating the hypothalamic–pituitary–adrenal (HPA) axis [63]; thirdly, exercise regulates intestinal pH and intestinal hormone release by affecting bile acid metabolism within the enterohepatic circulation [64], or may interact directly or indirectly with the intestine by stimulating the release of multiple myokines and metabolites [65].These exercise-induced intestinal adaptations affect the gut environment in a way that may select the surviving microorganisms, leading to alterations to gut microbiota composition [63,66]. However, further studies are still needed to determine the exact mechanisms by which exercise alters intestinal microbes, as well as the specific effects of exercise on certain bacteria.

A further analysis of intestinal microorganisms in the ADC group mice revealed that the dominant differential species were *Bacilli*, *Erysipelotrichales*, *Erysipelotrichaceae*, and *Allobaculum*. Pei et al. [67] showed that *Erysipelotrichales* and *Erysipelotrichaceae* species are increased in abundance in the intestine of patients with hematological malignancies and can be used as markers in these patients. A surge in the abundance of *Erysipelotrichales* is associated with intestinal inflammation [68] and blocking its growth in a model of parenteral nutrition-associated liver disease attenuates liver injury [69]. Hence, *Erysipelotrichales* and *Erysipelotrichaceae* are harmful bacteria associated with intestinal flora disorders and inflammation. It has been shown that *Allobaculum* species were significantly increased in a diabetic model group compared with a normal group [70,71]. Interestingly, AD has many similarities to diabetes and is even gradually being considered as type 3 diabetes [72]. It is evident that the decline in the diversity of the intestinal microbiota in AD mice leads to an overgrowth of harmful bacteria such as *Erysipelotrichaceae* and *Allobaculum*. The dominant differential species in the ADE group is *Akkermansia*, which has been proposed as a marker of a healthy intestine due to its anti-inflammatory and anti-immunostimulatory properties, as well as its ability to improve the intestinal barrier [73]. Our data also suggest that exercise may protect the intestinal barrier by upregulating the abundance of Akkermansia. In summary, treadmill exercise can modulate intestinal microbial diversity in AD mice, reducing the abundance of harmful bacteria (*Allobaculum*) and increasing that of beneficial bacteria (*Akkermansia*). However, further studies are needed to confirm whether *Akkermansia* plays a major role in conferring the beneficial effects of exercise on the intestinal barrier.

Clinical autopsies performed on AD patients have shown that hippocampal LPS levels are three times higher in AD patients than in age-matched control subjects, while hippocampal LPS levels are 26 times higher in late AD patients than in age-matched control subjects [74,75]. Our results showed that serum and brain LPS levels are significantly increased in AD mice, while exercise downregulates serum and brain tissue LPS levels. AD is characterized by severe intestinal and brain leakage, leading to LPS translocation; however, exercise can protect the intestinal barrier and BBB, suppressing LPS translocation. This result could attenuate the inflammatory cascade response at its source. Microglia, known as immunoreactive cells in the brain, are the most important players in the development and progression of neuroinflammation [76]. In our research, the immunofluorescent labeling of microglia revealed a significant abnormal aggregation in the cerebral cortex of AD mice, which could be reduced by exercise; however, whether this is associated with a decrease in Aβ and LPS levels needs to be determined through further investigation. TNF−α and IL−1β released from microglia due to activation are the main cytokines that induce neuroinflammation, which not only leads to cell activation and proinflammatory factor release that amplify inflammation in a continual cascade but also induces the activation of astrocytes through the classical NF−κB pathway, in turn leading to the production of reactive oxygen species (ROS) and NO and the release of large amounts of inflammatory factors, making astrocytes the major contributor to neuroinflammation [77,78,79,80]. Experiments have shown that astrocytes in the cerebral cortex of AD mice are abnormally activated and accumulate more prominently than microglia. Astrocytes are involved in controlling blood perfusion in the brain, maintaining BBB stability, and regulating neurons, with the function of maintaining the integrity of the central nervous system [81]. BBB catabolism has been observed in the elderly and in late-onset AD, and it is inextricably linked to neuroinflammation [82]. Cai et al. [83] noted that astrocyte dysfunction can easily impair the normal physiological function of the BBB and induce an imbalance in the clearance of Aβ from the brain parenchyma to the blood. In summary, in our experiments, we observed that treadmill exercise significantly reduces the abnormal activation of astrocyte aggregation in the cerebral cortex of AD mice, which could improve BBB function and maintain the tightness of the physical barrier while maintaining the transporter protein transport barrier and mitigating the risk of neuroinflammation and Aβ deposition.

Interestingly, Kahn et al. [84] injected LPS into the peritoneal cavity of mice and found an increased transfer of Aβ from the blood to the brain and a decreased transfer from the brain to the blood, while the production of Aβ by neurons was exacerbated. This suggests that LPS may be the source of neuroinflammation and Aβ deposition in AD and, therefore, are an important target molecule contributing to AD pathology and exacerbating the AD process. In summary, we suggest that chronic systemic inflammation increases progressively as the body ages, and intestinal microbial disorders exacerbate the leakiness of intestines, leading to increased LPS production and the displacement of LPS to the internal environment and further damage to the BBB. Moreover, LPS entering the brain exacerbate the neuronal production of Aβ and activate glial cells, resulting in a series of AD pathologies. Exercise has a systemic effect and can simultaneously regulate all aspects of the microbe–gut–brain axis, preventing the harmful molecules from entering the brain and interrupting or slowing down the vicious cycle of LPS–neuroinflammation–neurodegeneration, resulting in significant benefits towards the alleviation and prevention of AD. We explored the peripheral mechanisms of AD neuroinflammation from the perspective of the microbe–gut–brain axis and found that exercise may alleviate AD neuroinflammation by modulating intestinal microbiota and suppressing leaky intestine immune activation, suggesting that further research into these mechanisms is worthwhile. However, the role of LPS in the reduction of inflammatory factors in the brain by exercise must be confirmed by further studies, because there are other causes of inflammation in AD such as Aβ. Aβ, microglia, and astrocytes play an important role in the pathogenesis of AD, but the causal relationship between them and LPS has yet to be explored by comprehensive systematic testing and deserves further study in a follow-up. In addition, it was found that exercise did not significantly contribute to the regulation of learning and memory capacity, as well as intestinal and cerebral regulation, in wildtype mice in contrast to AD mice; this result is similar to the results of Yan et al. [85], who revealed that exercise seemingly had more of a more corrective effect on abnormal organisms than an effect on improving normal organisms. Further investigations are needed to determine whether this observation is related to the exercise intensity, age, and strain of the animals.

## 5. Conclusions

**Figure 8:**
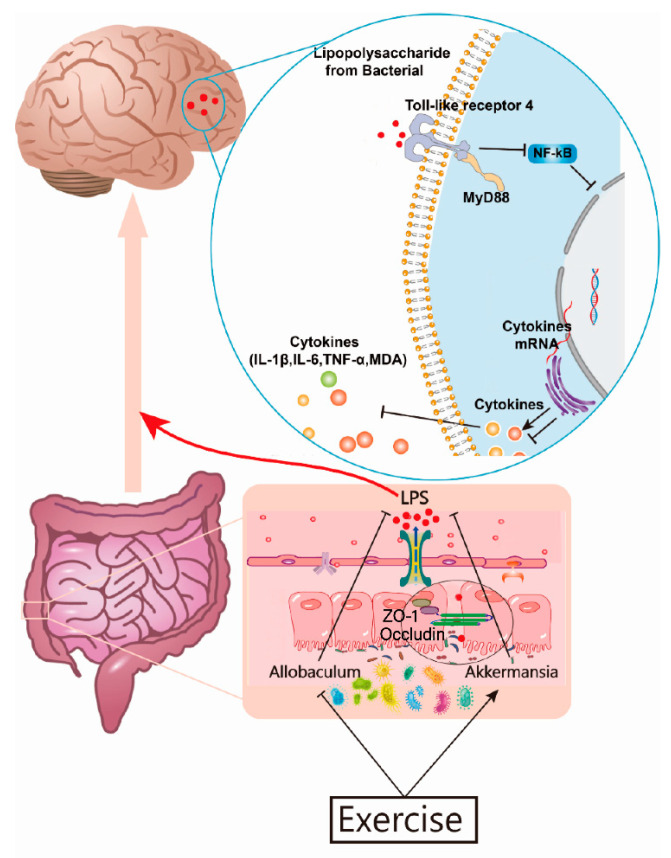
Possible mechanism through which exercise alleviates neuroinflammation in the brains of AD mice.

In conclusion, treadmill exercise alleviates brain neuroinflammation and spatial memory capacity in APP/PS1 mice and enriches intestinal microbial diversity, protecting the blood–brain barrier via the intestinal barrier, and suppressing the translocation of LPS to the brain. A potential mechanism for the beneficial effects of exercise on AD is illustrated in Figure 8. It is hypothesized that intestinal *Akkermansia* may be an important target bacterium and LPS may be important peripheral target molecules by which exercise confers improvements in the context of AD.

## Figures and Tables

**Table 1:** Primer sequences of target genes.

Gene.	Forward Primer	Reverse Primer
TLR4	ACAAACGCCGGAACTTTTCG	GTCGGACACACACAACTTAAGC
MyD88	TCATGTTCTCCATACCCTTGGT	AAACTGCGAGTGGGGTCAG
TNF−α	TTAGAAAGGGGATTATGGCTCA	ACTCTCCCTTTGCAGAACTCAG
IL−6	CATTTCCACGATTTCCCAGA	CGGAGAGGAGACTTCACAGAG
IL−1β	CTCACAAGCAGAGCACAAGC	AGCTGTCTGCTCATTCACGA
GAPDH	CATGGCCTTCCGTGTTCCTA	CCTGCTTCACCACCTTCTTGAT

**Table 2:** The values of α-diversity indices for intestinal microbes of different groups of mice.

Group	Sobs	Shannon	Simpson	ACE	Chao
WTC	566.5	3.746	0.059	768.326	720.746
WTE	684.5	4.242	0.032	852.780	829.374
ADC	473.833	3.695	0.066	614.769	578.869
ADE	657.667	4.012	0.051	824.108	811.765
